# Supplementary figures and images for: Inhibition of circulating dipeptidyl-peptidase 3 restores cardiac function in a sepsis-induced model in rats: A proof of concept study
Source: PLoS One. 2020 Aug 27;15(8):e0238039. doi: 10.1371/journal.pone.0238039 (PMC7451654; doi:10.1371/journal.pone.0238039)

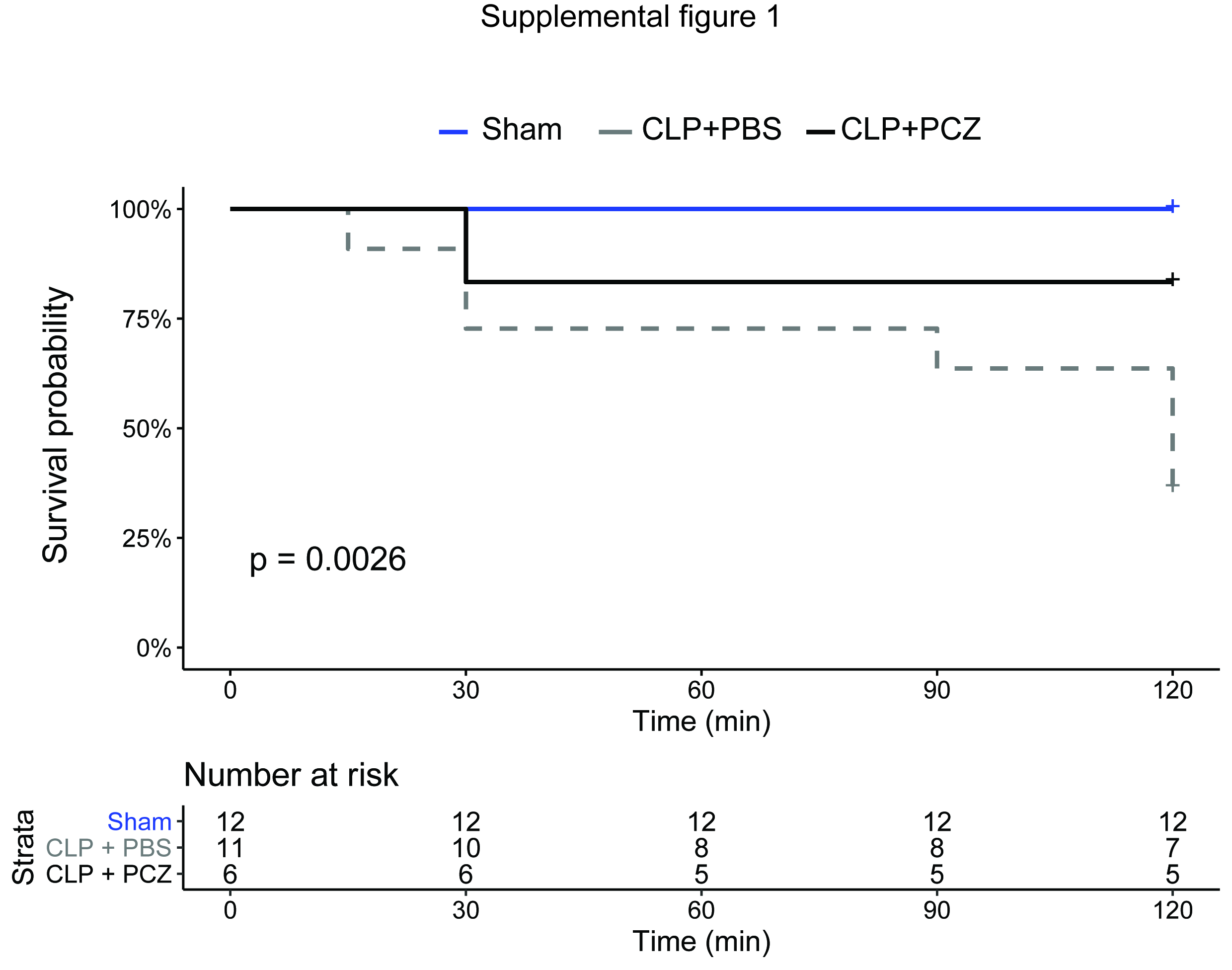

Supplement: S1 Fig — CLP: cecal ligation and puncture, PBS: phosphate buffered saline, PCZ: Procizumab. Log rank test was used. (TIF) [file pone.0238039.s001.tif]

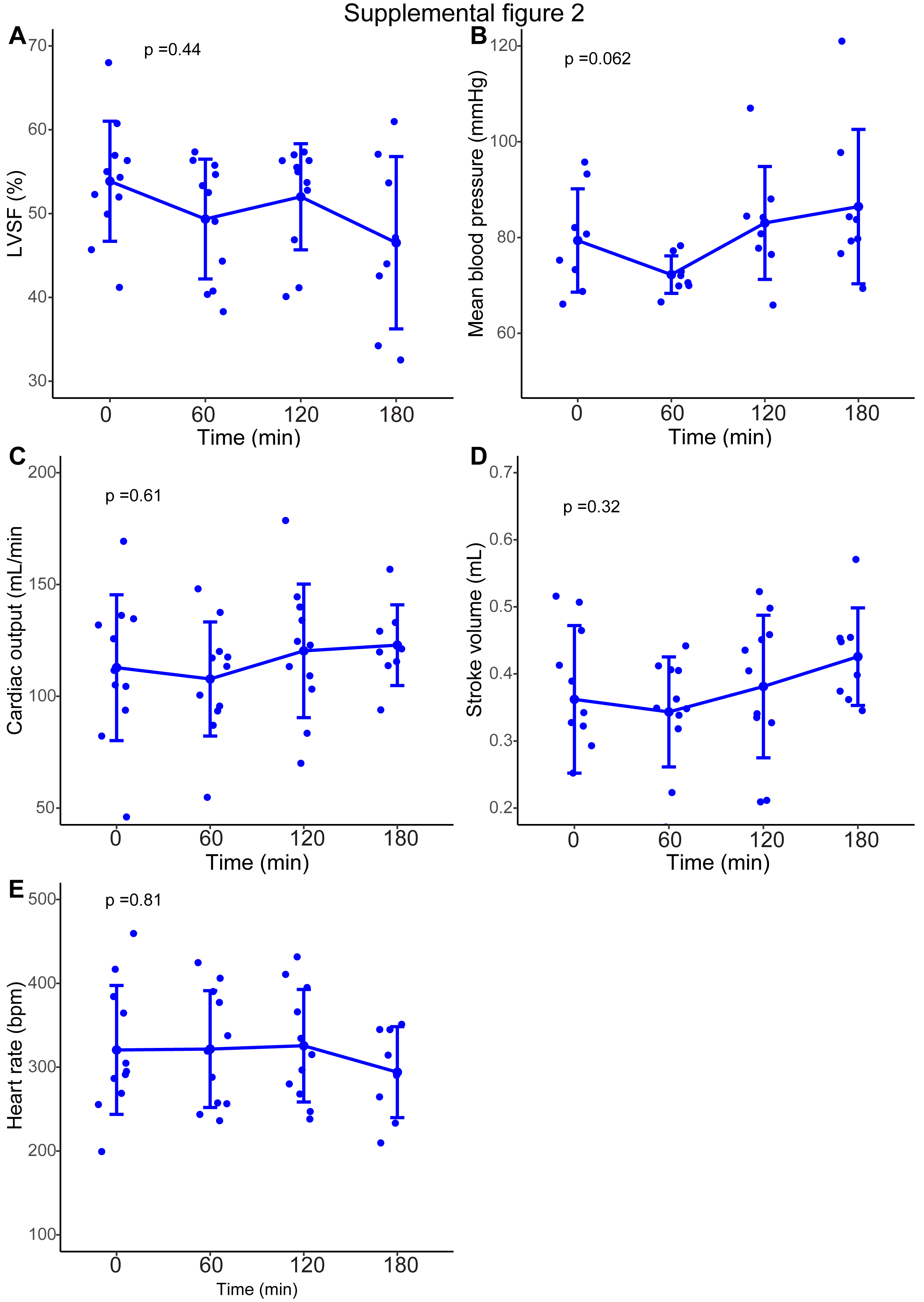

Supplement: S2 Fig — Schematic representation of the evolution of the left ventricular shortening fraction (A), mean blood pressure (B), cardiac output (C), stoke volume (D) and heart rate (E) 16 hours after sham surgical procedure. Comparisons were made by using repeated measures of ANOVA. (TIF) [file pone.0238039.s002.tif]
