# Supplementary material for: Inhibition of circulating dipeptidyl-peptidase 3 restores cardiac function in a sepsis-induced model in rats: A proof of concept study
Source: PLoS One. 2020 Aug 27;15(8):e0238039. doi: 10.1371/journal.pone.0238039 (PMC7451654; doi:10.1371/journal.pone.0238039)
Supplement: S1 Table — (DOCX) [file pone.0238039.s003.docx]

**Supplemental table 1**

| **Name** | **Gene** | **Access GenBank** | **Forward sequence** | **Reverse sequence** |
| --- | --- | --- | --- | --- |
| **GAPDH** | Gapd | NM_017008.4 | GTTCAACGGCACAGTCAAGG | ACTCCACGACATACTCAGCAC |
| **DPP3** | Dpp3 | NM_053748.1 | GGAACCAAGGCTTCGACATC | TGAAGAGCCGGGTGTTGTAT |
| **HO1** | Hmox1 | NM_012580.2 | TGCTGACAGAGGAACACAAAGA | CGGTCGCCAACAGGAAACT |
| **NQO1** | Nqo1 | NM_017000.3 | ACCTTGCTTTCCATCACCACC | GACGCTTCTTCCACCCTTCC |

Supp. Table 1.
